# Characterization, Quantification, and Determination of the Toxicity of Iron Oxide Nanoparticles to the Bone Marrow Cells

**DOI:** 10.3390/ijms160922243

**Published:** 2015-09-14

**Authors:** Sae-Yeol-Rim Paik, Jong-Seok Kim, Sung Jae Shin, Sanghoon Ko

**Affiliations:** 1 Department of Food Science and Technology, Sejong University, 209 Neungdong-ro, Gwangjin-gu, Seoul 143-747, Korea; E-Mail: serim0506@naver.com; 2 Department of Microbiology and Institute for Immunology and Immunological Diseases, Yonsei University College of Medicine, 50 Yonsei-ro, Seodaemun-gu, Seoul 120-752, Korea; E-Mails: jskim7488@yuhs.ac (J.-S.K.); sjshin@yuhs.ac (S.J.S.)

**Keywords:** iron nanoparticle, physicochemical property, cytotoxicity, bone marrow cell

## Abstract

Iron oxide nanoparticles (IONPs) have been used to develop iron supplements for improving the bioavailability of iron in patients with iron deficiency, which is one of the most serious nutritional deficiencies in the world. Accurate information about the characteristics, concentration, and cytotoxicity of IONPs to the developmental and reproductive cells enables safe use of IONPs in the supplement industry. The objective of this study was to analyze the physicochemical properties and cytotoxicity of IONPs in bone marrow cells. We prepared three different types of iron samples (surface-modified iron oxide nanoparticles (SMNPs), IONPs, and iron citrate) and analyzed their physicochemical properties such as particle size distribution, zeta potential, and morphology. In addition, we examined the cytotoxicity of the IONPs in various kinds of bone marrow cells. We analyzed particle size distribution, zeta potential, iron levels, and subcellular localization of the iron samples in bone marrow cells. Our results showed that the iron samples were not cytotoxic to the bone marrow cells and did not affect the expression of cell surface markers and lipopolysaccharide (LPS)-induced the secretion of cytokines by murine bone marrow-derived dendritic cells (BMDCs). Our results may be used to investigate the interactions between nanoparticles and cells and tissues and the developmental toxicity of nanoparticles.

## 1. Introduction

Iron is an essential mineral supplement for all living organisms. In addition, iron has several important functions in the human body. Iron is a key component of the hemoglobin protein that carries oxygen to the tissues in the blood. Iron deficiency is one of the most common nutritional deficiencies in the world. The risk of iron deficiency or iron deficiency anemia is normally high in pregnant woman, pre-menopausal women, children, and people with a poor diet [1]. While untreated iron deficiency can cause complications in pregnant women and cause delayed growth and behavior disturbances in children, high levels of iron in the blood can damage proteins (enzymes), DNA, and other components because it reacts with peroxides to produce free radicals [1,2]. Iron supplements are used to treat iron deficiency and iron deficiency anemia. However, iron supplements have low bioavailability and may be associated with gastrointestinal side effects. Therefore, development of a new iron supplement with high bioavailability and less side effects is required.

Recently, nanotechnology has been applied to the foods, nutrition supplements, and pharmaceutics since nanotechnology-applied materials are considered highly bioaccessible and bioavailable in the gastrointestinal tract [3,4,5,6,7,8]. Iron oxide nanoparticles (IONPs) can be used to overcome the shortcomings associated with iron supplements because IONPs have a large specific surface area, which can improve the bioavailability of iron [9]. Moreover, low doses of IONPs are required, and thus, the risk of side effects and the cost of treatment are decreased. However, because of the high bioavailability, an optimum dosage of the IONPs should be established in order to avoid side effects or toxicity due to high levels of iron. Therefore, for the application of iron nanoparticles in the supplement industry, we need to examine and understand the bioavailability, developmental and reproductive toxicity, and long-term effects of iron nanoparticles. Moreover, a suitable standard protocol for the physicochemical characterization and quantification of iron nanoparticles should be established.

There have been a number of biological studies on nanoparticles during the last couple of decades, and the concerns about the impact of nanoparticles on public health have increased because of their potential toxicity [4]. Typically, the toxicity of nanomaterials depends on their physicochemical properties such as particle size, surface properties, and chemical composition [10,11]. Therefore, the physicochemical characteristics must be accurately determined before assessing the *in vitro* and *in vivo* toxicity of nanoparticles. To date, many studies have reported the toxicity of iron oxide nanoparticles. While some studies report that IONPs are non-toxic [12,13], many studies indicate that further studies are required to clarify the toxicity of nanoparticles [14,15].

The physicochemical properties of nanoparticles such as particle size and zeta potential are important for understanding the interaction of nanoparticles with biological systems [16]. Because of the limited number of studies to date on the physicochemical characterization of nanoparticles in biological systems, understanding the interaction of nanoparticles with biological systems such as developmental and reproductive tissues is difficult. Therefore, development of a standard method to analyze nanoparticles in biological systems is urgently required.

Bone marrow cells produce red blood cells, white blood cells, and fat cells in the human body. In addition, bone marrow is a key component of the lymphatic system that supports the immune system. A method for analyzing nanoparticles in bone marrow cells could improve the understanding of the effects of nanoparticles on cellular development and on the immune system. Determination of the toxicity of IONPs based on their physicochemical properties can enable the use of IONPs in various fields. Iron nanoparticles can be used safely in various forms such as nutrient supplements, imaging agents in magnetic resonance and magnetic particle imaging, and as photocatalysts.

The objective of this study is to establish a method for analyzing the physicochemical properties and cytotoxicity of IONPs in bone marrow cells. We prepared three different types of iron samples (surface-modified iron oxide nanoparticles (SMNPs) IONPs, and iron citrate) and analyzed their physicochemical properties such as particle size distribution, zeta potential, and morphology. In addition, we examined the cytotoxicity of the IONPs in various kinds of bone marrow cells.

## 2. Results and Discussion

### 2.1. Preparation of SMNPs (Surface-Modified Iron Oxide Nanoparticles)

Highly dispersible SMNPs were prepared by suspending insoluble IONPs in deionized water and treating them using a citrate-functionalization method [16]. IONPs have a large specific surface area, and thus, they tend to quickly agglomerate to thermodynamically decrease the Gibb’s free energy among the particles [17,18]. Therefore, in aqueous media, the SMNPs solubilized using the citrate-functionalization are monodisperse, whereas IONPs may be present as agglomerates.

Accurately measuring the primary particle size distribution of mineral nanoparticles has been a challenge to the preparation of monodisperse suspension, such as IONPs, for the last few decades [16]. Monodispersity of the mineral nanoparticles has been achieved using several techniques such as surface modification, micellization, coating, *etc.* [19,20]. The difference in the surface characteristics of the mineral nanoparticles may strongly affect the cytotoxicity of nanoparticles. Therefore, a proper surface modification of IONPs is needed before using them in the cytotoxicity studies. Herein, the modification of surface charge of IONPs with the mixture of citric acid and sodium citrate was chosen since they are recognized as non-toxic and biocompatible materials. Thus, we focused on the investigation of the effect of IONPs on the cytotoxicity, whereas we tried to minimize the effect of the surface modification.

### 2.2. Characterization of IONPs (Iron Oxide Nanoparticles) and SMNPs

#### 2.2.1. Particle Size Distribution

**Figure 1:**
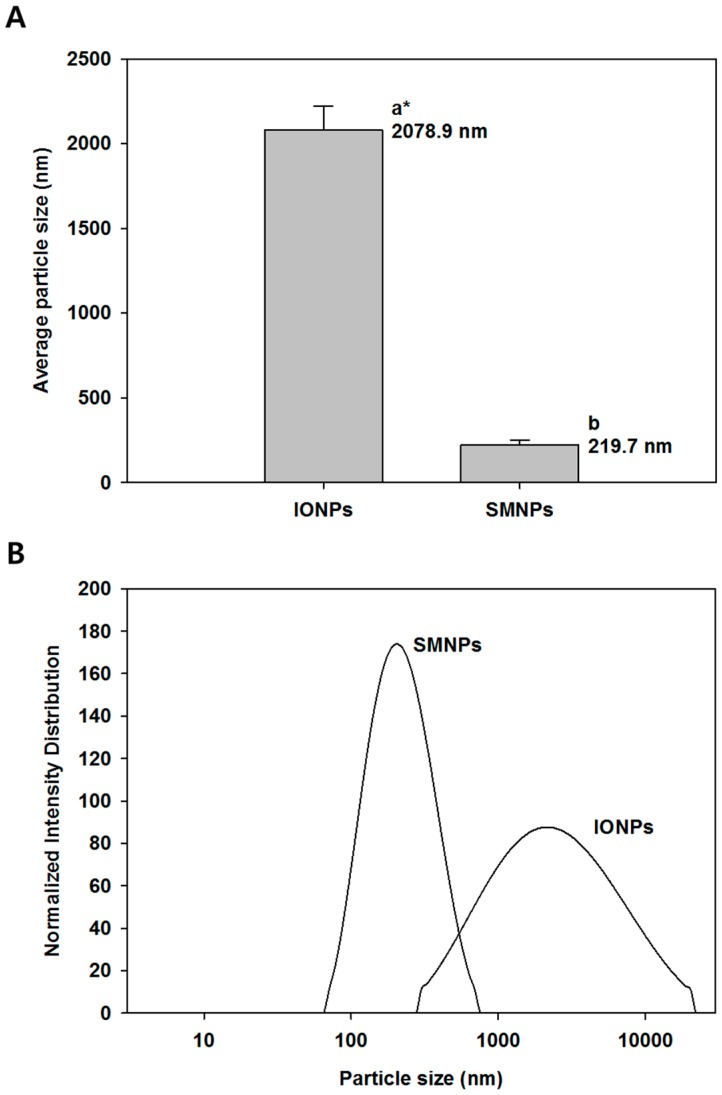
Particle size and size distribution of surface-modified nanoparticles (SMNPs) and iron oxide nanoparticles (IONPs): average particle size (**A**) and normalized size distribution based on intensity of the scattered light (**B**). SMNPs were prepared by surface modification of the IONPs using a citrate functionalization method [16]. * Different letters indicate significant differences in mean values (*p* < 0.05).

The average particle size and the size distribution of IONPs and SMNPs were measured using a dynamic laser light-scattering particle size analyzer. The average particle size of IONPs and SMNPs is shown in Figure 1A; the average particle size of IONPs and SMNPs were 2078.9 and 219.7 nm, respectively. The SMNPs showed narrower particle size distribution and smaller average particle size. However, the particle size data obtained using the dynamic laser light-scattering particle size analyzer is not the average size of individual IONPs since insoluble nanoparticles such as IONPs are commonly agglomerated or precipitated in aqueous media, which result in an incorrect particle size. The particle size data of SMNPs measured using the dynamic laser light-scattering particle size analyzer is reliable because SMNPs have a citrate group on their surface, which is highly dispersible in the aqueous medium [16]. Our results indicated that the size of surface-modified particles was smaller than that of non-modified particles. In addition, the size distribution of SMNPs is narrower than that of IONPs (Figure 1B).

#### 2.2.2. Zeta Potential

**Figure 2:**
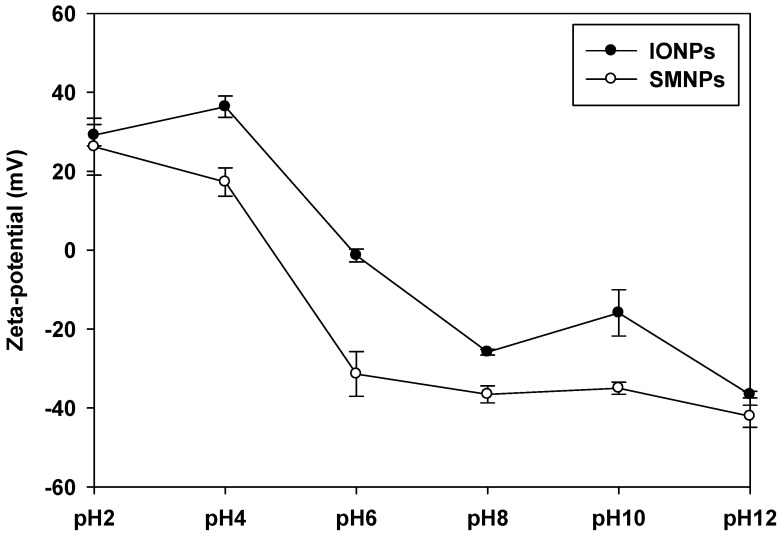
Zeta potential of surface-modified nanoparticles (SMNPs) and iron oxide nanoparticles (IONPs). SMNPs were prepared by surface modification of the IONPs using a citrate functionalization method [16].

The zeta potential profiles of nanoparticles at different pH are shown in Figure 2. At neutral pH, the value of the zeta potential of SMNPs (approximate −35 mV) was lower than that of IONPs (approximate −20 mV). The zeta potential value far from zero indicates that the particles disperse well in the media since the electrostatic repulsive force among the particles is large, and thus, the particles have high aqueous stability. Thus, the nanoparticle dispersion with a value of zeta potential far from zero was stable or relatively monodisperse, while that with a value close to zero indicated poor monodispersity [21].

The dispersion of SMNPs at pH 6 was more stable than that of IONPs (Figure 2). On the contrary, IONPs had a low surface charge, which resulted in instability in an aqueous medium. The particles with the zeta potential close to zero agglomerate to minimize Gibb’s free energy. Surface modification by citrate functionalization could prevent particle agglomeration and enable dispersion of individual particles in the media. Therefore, the difference in the stability of IONPs and SMNPs affects cytotoxicity.

### 2.3. Iron Levels in the Bone Marrow Cells

**Figure 3:**
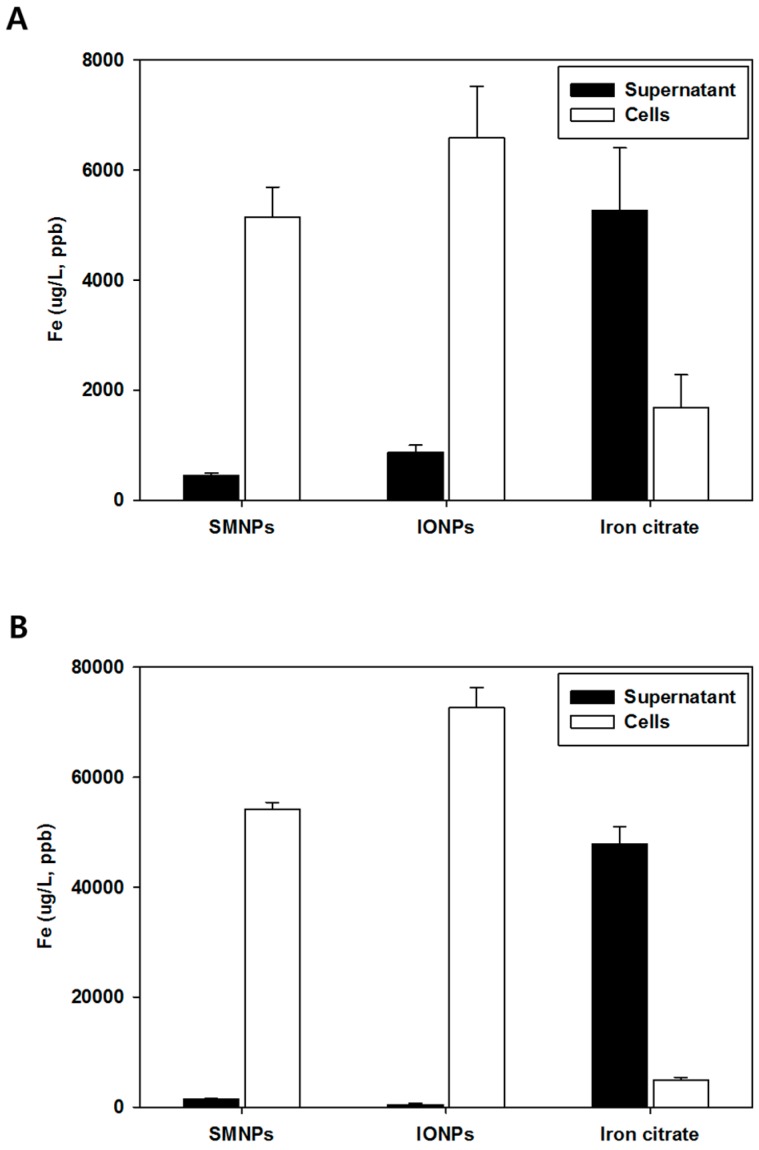
Iron levels of surface-modified nanoparticles (SMNPs), iron oxide nanoparticles (IONPs), and iron citrate in the bone marrow cells and the cell supernatants. The cells were treated with 1% (**A**) and 10% (**B**) iron. SMNPs were prepared by surface modification of the IONPs using a citrate functionalization method [16].

The iron concentrations after incubation with 1% of SMNPs, IONPs, and iron citrate are shown in Figure 3A. Most of the iron from the SMNPs and IONPs samples was found in the cells (pellet). However, the iron from iron citrate was found in the supernatant, which indicated no interactions between the bone marrow cells and iron citrate. The SMNPs and IONPs may have undergone phagocytosis during incubation with bone marrow cells. Results similar to those reported above were obtained using 10% of SMNPs, IONPs, and iron citrate. The total concentrations of iron in 1% and 10% IONPs were approximate 7000 and 60,000 μg/L, respectively.

**Figure 4:**
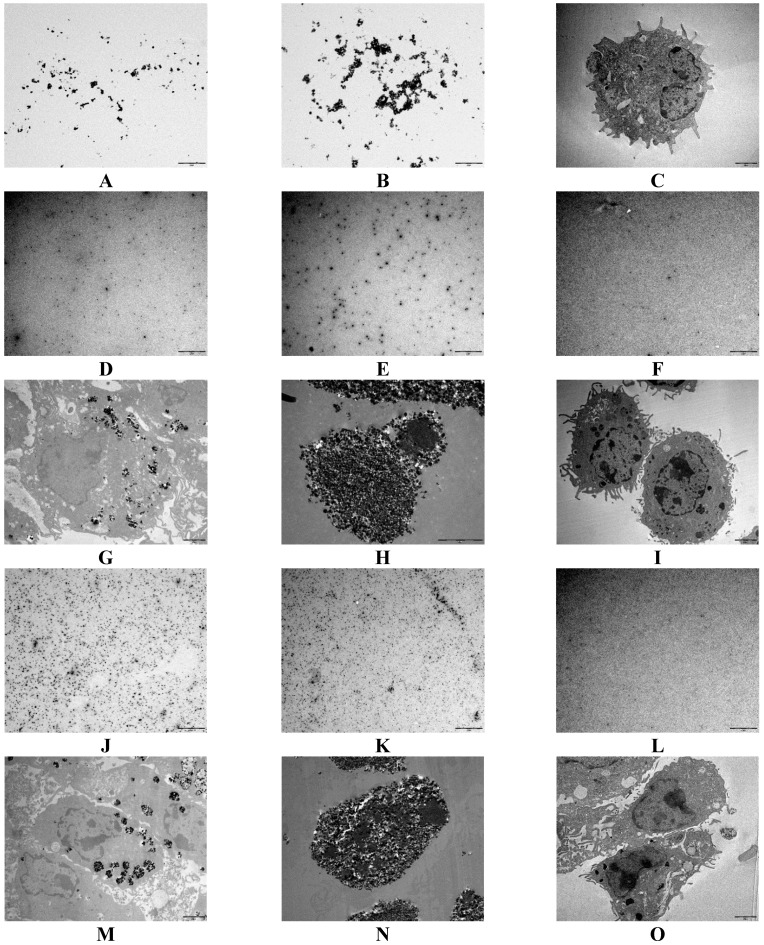
Morphology and subcellular localization of surface-modified nanoparticles (SMNPs), iron oxide nanoparticles (IONPs), and iron citrate in bone marrow cells. SMNPs (**A**); IONPs (**B**); control bone marrow cells (**C**); cell supernatant with 1% SMNPs (**D**); cell supernatant with 1% IONPs (**E**); cell supernatant with 1% iron citrate (**F**); cell pellet with 1% SMNPs (**G**); cell pellet with 1% IONPs (**H**); cell pellet with 1% iron citrate (**I**); cell supernatant with 10% SMNPs (**J**); cell supernatant with 10% IONPs (**K**); cell supernatant with 10% iron citrate (**L**); cell pellet with 10% SMNPs (**M**); cell pellet with 10% IONPs (**N**); and cell pellet with 10% iron citrate (**O**). Scale bars in the images indicate 2 μm.

We obtained transmission electron micrography (TEM) images of SMNPs and IONPs to determine the particle shape and size. The dried SMNPs and IONPs are shown in Figure 4A,B. Although we observed agglomeration of IONPs, the shape and individual particle size of SMNPs and IONPs were same.

To determine the subcellular localization of iron nanoparticles, we used TEM images shown in Figure 4C–O. Bone marrow cells without nanoparticles were used as a control. We examined the subcellular localization using 1% and 10% iron concentrations. Treatment with 1% of SMNPs, IONPs, and iron citrate is shown in Figure 4D–I while that with 10% of SMNPs, IONPs, and iron citrate is shown in Figure 4J–O. Some small nanoparticles did not sink, and the IONPs were larger than SMNPs (Figure 4D,E). In addition, the supernatant of cells treated with iron citrate did not show any iron particles (Figure 4F). The TEM images of fixed bone marrow cells with SMNPs, IONPs, and iron citrate are shown in Figure 4G–I, respectively. In these images, dark black spots indicate iron particles. Some iron particles from the SMNPs were observed in the bone marrow cells and the cell membrane did not appear to be damaged. However, a large number of iron particles from the IONPs were detected in the cell culture, and the membranes of most of the cells were damaged. Further, we were unable to observe differences between the TEM images of control (bone marrow cells) and cells with iron citrate. Thus, iron salts did not affect the bone marrow cells. Moreover, iron citrate cannot be detected in the bone marrow cells by TEM without using a labeling agent or a dye. The same results as those reported above were observed using the samples with 10% iron concentration (Figure 4J–O). However, we observed more number of particles using samples containing 10% iron than those observed using 1% iron.

The TEM images indicate that cells treated with IONPs had higher levels of iron than those treated with SMNPs. Bone marrow cells treated with IONPs contained more iron particles. Thus, the effect of phagocytosis was stronger in IONPs than in SMNPs. Our results indicate that the agglomerated iron nanoparticles can affect cells and surface modification can reduce the cellular toxicity of iron nanoparticles.

### 2.4. Effect of SMNPs, IONPs, and Iron Citrate on the Cytotoxicity of Various Kinds of Eukaryotic Cells

**Figure 5:**
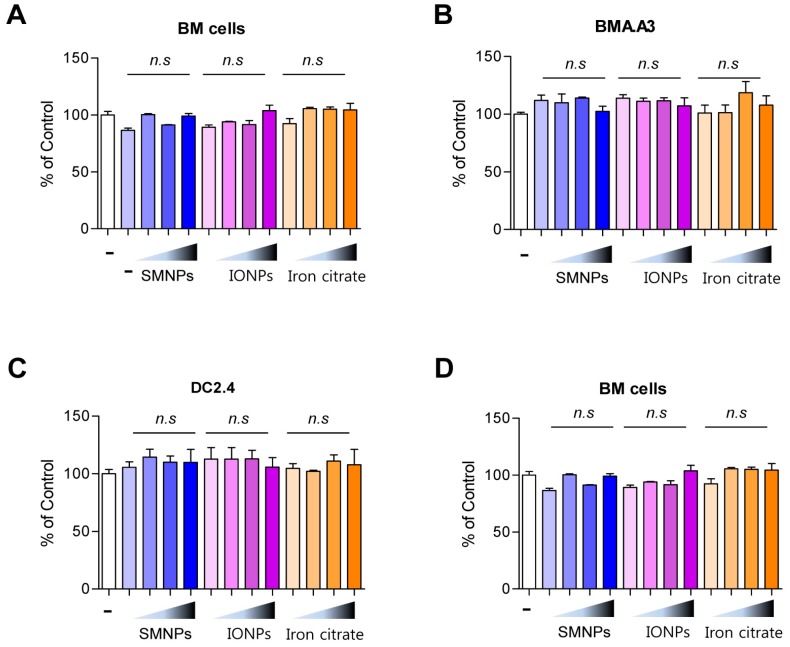
Cytotoxicity of surface-modified nanoparticles (SMNPs), iron oxide nanoparticles (IONPs), and iron citrate to DC2.4, BMA.A3, A549, and mouse bone marrow cells. Mouse bone marrow cells (**A**); BMA.A3 (**B**); DC2.4 (**C**); and A549 cells (**D**) were plated in 96-well culture plates (1 × 10^4^ cells/well). After 24 h, the cells were incubated with 0.01%, 0.1%, 1% and 10% concentrations (*w*/*v*) of SMNP, IONP, and iron citrate, respectively, for 72 h. Cytotoxicity was evaluated using CCK-8 as described in the Experimental Section (n.s, no significant).

To investigate the effect of SMNPs, IONPs, and iron citrate on dendritic differentiation, we examined the cytotoxicity of SMNPs, IONPs, and iron citrate on murine bone marrow cells. SMNPs, IONPs, and iron citrate were not cytotoxic to bone marrow cells when used at concentrations below 1% (Figure 5A). Further, to determine whether the effects of SMNPs, IONPs, and iron citrate were specific to bone marrow cells, we examined the cytotoxicity of these samples on DC2.4 murine dendritic cells, BMA.A3 murine macrophages, and A549 human lung carcinoma epithelial cells. Incubation with SMNPs, IONPs, and iron citrate for 72 h did not have any toxic effects on these three different cell lines (Figure 5B–D). These results indicated that SMNPs, IONPs, and iron citrate were not cytotoxic to murine bone marrow and three other types of cells.

### 2.5. Effect of SMNPs, IONPs, and Iron Citrate on the Differentiation of Murine Bone Marrow-Derived Dendritic Cells

**Figure 6:**
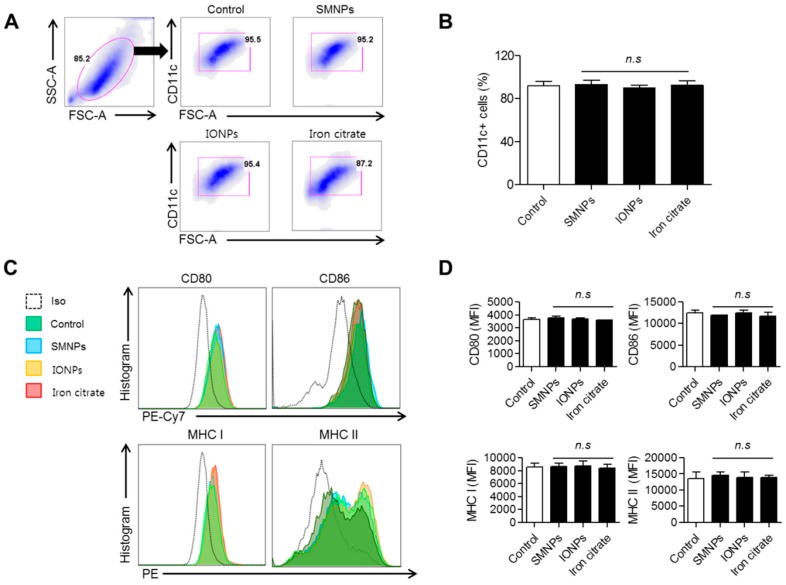
Effects of surface-modified nanoparticles (SMNPs), iron oxide nanoparticles (IONPs) and iron citrate on differentiation of bone marrow dendritic cells (BMDCs). Bone marrow cells were isolated and incubated with RPMI1640 media containing 20 ng/mL granulocyte-macrophage colony stimulating factor (GM-CSF), 5 ng/mL interleukin 4 (IL-4), and 10% fetal bovine serum (FBS) for six days. During incubation, the bone marrow cells were treated with 1% of SMNPs, IONPs, and iron citrate. After six days, the BMDCs were stained with anti-CD11c monoclonal antibody (mAb). (**A**) BMDCs were primary gated on FSCmid/low and SSCmid/low; (**B**) Bar graphs show the mean ± standard error of mean (SEM) of percentage of CD11c-positive cells representing three independent experiments (n.s, no significant); (**C**) BMDMs were stained with anti-CD80, anti-CD86, anti-MHC class I, or anti-MHC class II antibodies; (**D**) Bar graphs show the mean ± SEM of percentage of each surface molecule on CD11c-positive cells representing three independent experiments (n.s, no significant).

To investigate whether SMNPs, IONPs, and iron citrate affect the differentiation of dendritic cells, we added these samples during the differentiation of dendritic cells. After six days, we compared the number of CD11c-positive cells, which is a well-known marker of dendritic cells. We observed no significant difference among the treated cells (Figure 6A,B).

In addition, we analyzed various cell-surface markers, including CD80, CD86, MHC class I, and MHC class II on murine BMDCs. Treatment with SMNPs, IONPs, and iron citrate did not affect the expression of cell-surface markers (Figure 6C,D).

### 2.6. Effect of SMNPs, IONPs, and Iron Citrate on the Secretion of Cytokines by BMDCs

**Figure 7:**
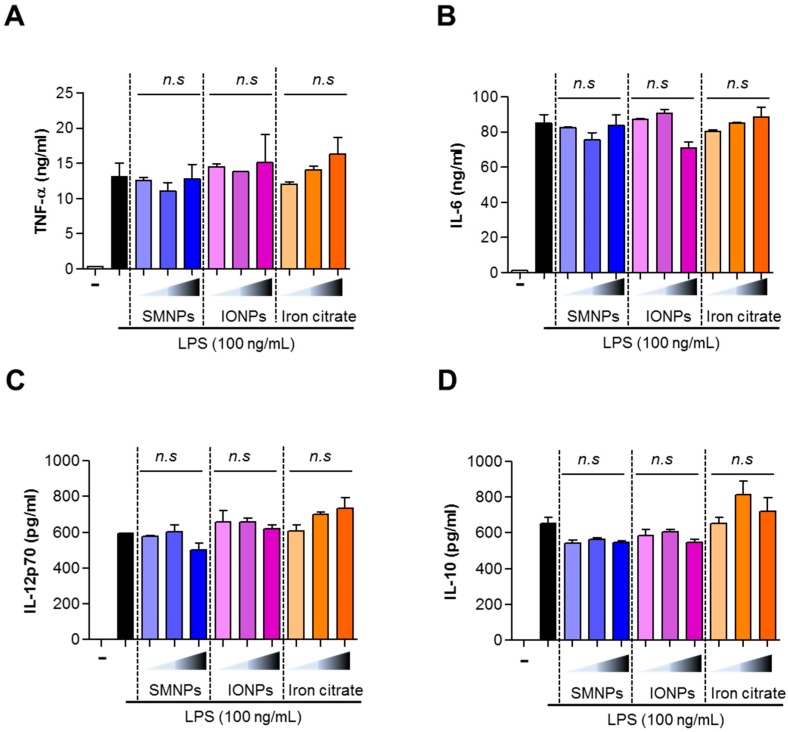
Effects of surface-modified nanoparticles (SMNPs), iron oxide nanoparticles (IONPs), and iron citrate on the production of cytokines in lipopolysaccharide (LPS)-stimulated bone marrow dendritic cells (BMDCs). BMDCs were stimulated with 100 ng/mL LPS in absence or presence of 1% of SMNP, IONP, and iron citrate for 24 h. We measured the levels of tumor necrosis factor α (TNF-α) (**A**), interleukin 6 (IL-6) (**B**), IL-12p70 (**C**), and IL-10 (**D**) in the culture supernatants by using ELISA. All data were expressed as the mean ± standard deviation (SD) (*n* = 6) (n.s, no significant).

Stimulation of Toll-like receptor 4 (TLR4) by LPS induces maturation of DCs and secretion of proinflammatory cytokines. To investigate the effects of the iron samples on the secretion of cytokines by BMDCs, we treated the BMDCs with SMNPs, IONPs, and iron citrate and LPS for 24 h and measured the levels of tumor necrosis factor α (TNF-α), interleukin 6 (IL-6), IL-12p70, and IL-10. Treatment with SMNPs, IONPs, and iron citrate did not affect LPS-induced secretion of cytokines by BMDCs (Figure 7A–D).

## 3. Experimental Section

### 3.1. Materials

IONPs (Fe_2_O_3_, FE-OX-03-NP, purity 99.9%) were commercially supplied by American Elements (Los Angeles, CA, USA). Analytical grade citric acid, sodium citrate, and carboxymethyl cellulose (CMC) were supplied by Daejung Chemical & Metals Co. (Shiheung, Korea). Other chemicals were supplied by Sigma-Aldrich (St. Louis, MO, USA).

### 3.2. Preparation of SMNPs

SMNPs were prepared by surface modification of the IONPs using a citrate functionalization method [16]. We dispersed 2 g of IONPs in 100 mL of deionized water, and the dispersion was stirred at 500 rpm for 30 min. Next, we added 0.2 mL of 0.1 M citric acid, 0.4 mL of 0.1 M sodium citrate, and 50 mg of CMC to the IONPs dispersion, whose pH value was adjusted to 5.5, and the dispersion was stirred at 500 rpm for 30 min. Then, the dispersion was sonicated at 38% of the amplitude power for 10 min. The SMNPs prepared were stored and used for further study.

### 3.3. Characterization of IONPs and SMNPs

#### 3.3.1. Particle Size Measurements Using Differential Light Scattering

A laser light-scattering particle size analyzer (DelsaNano, Beckman Coulter, Fullerton, CA, USA) was used to measure the average particles size and size distribution of IONPs and SMNPs. The particle sizes were measured at 25 °C using a laser (wavelength, 632.8 nm) at a scattering angle of 165°. The average particle sizes were determined using the intensity of the scattered light. The particle sizes of all samples were measured at least three times.

#### 3.3.2. Measurement of Zeta Potential

The zeta potentials of IONPs and SMNPs were measured using a commercial zeta potential analyzer (DelsaNano, Beckman Coulter, Inc.). The dispersion was stirred continuously at 25 °C for 30 min before the measurements. The zeta potential measurements were performed in triplicate.

#### 3.3.3. TEM Measurements

TEM specimens were prepared by dropping a dilute dispersion of IONPs and SMNPs in phosphotungstic acid onto Formvar-coated grids with 200 square meshes. Each grid was held horizontally for 20 s at an angle of 45° to allow drainage of excess fluid. The nanoparticle specimens were air-dried before examination. The TEM specimens were examined using a transmission electron microscope (JEM-2011, Jeol Ltd., Tokyo, Japan). Monochromatic images of the nanoparticles were obtained as tagged in the images.

### 3.4. Cell Lines and Cell Culture

Human lung carcinoma epithelial cell line A549 was purchased from American Type Culture Collection (ATCC, Manassas, VA, USA). Murine dendritic cell line DC2.4 and murine macrophage-like cell line BMA.A3 were kindly provided by the Department of Biochemistry, Yonsei University, Seoul, Korea. All cells were cultured in Dulbecco’s modified Eagle’s medium DMEM (Biowest, Nuaillé, France) supplemented with 10% heat-inactivated fetal bovine serum (FBS, Biowest), 100 units/mL penicillin, and 100 μg/mL streptomycin at 37 °C in a humidified incubator containing 5% CO_2_.

### 3.5. Determination of Iron Levels

Inductively coupled plasma mass spectrometry (ICP-MS) was used to determine the iron content in the cell culture pellet and supernatant. Bone marrow cells were incubated with 1% and 10% of SMNPs, IONPs, and iron citrate. After incubation, all cell samples were centrifuged slightly. Subsequently, we collected the pellet and supernatant. Before the analysis, each sample was digested with nitric acid (HNO_3_) for 1 h at 65 °C on a heating block.

### 3.6. Generation and Culture of DCs

BMDCs from C57BL/6 mice were prepared and cultured as reported previously [22]. All animal experiments were performed in accordance with the Korean Food and Drug Administration (KFDA) guidelines. Briefly, the mice were euthanized by CO_2_ inhalation, bone marrow cells were plated in Petri dishes and cultured at 37 °C in the presence of 5% CO_2_ using RPMI 1640 media supplemented with 100 units/mL penicillin/streptomycin (Lonza, Basel, Switzerland), 10% FBS, 50 μM mercaptoethanol (Lonza), 20 ng/mL of recombinant mouse granulocyte-macrophage colony stimulating factor (GM-CSF, R&D Systems, Minneapolis, MN, USA), and 5 ng/mL IL-4 (R&D Systems). On Day 6, over 80% of the non-adherent cells expressed CD11c.

### 3.7. Analysis of Cytotoxicity

Cells were seeded at a density of 1 × 10^4^ per well in 96-well culture plates and treated with SMNPs, IONPs, and iron citrate for 3 days; cell viability was assessed using a Cell Counting Kit-8 (CCK-8, Dojindo Laboratory, Kumamoto, Japan). Aliquots of the kit reagent were added to the culture medium, and the plate was incubated at 37 °C for another hour. Cell viability was determined by measuring the absorbance at 450 nm by using a microplate reader.

### 3.8. Fluorescence-Activated Cell Sorting

Cell surface staining was performed with specifically labeled fluorescent-conjugated antibodies as reported previously [22]. BMDCs were cultured in RPMI1640 complete medium (10% FBS, 100 U/mL penicillin, and 100 μg/mL streptomycin) in 12-well culture plates that contained various concentrations of SMNPs, IONPs, and iron citrate or 100 ng/mL of LPS. The samples were incubated for 24 h at 37 °C in an incubator containing 5% CO_2_. Then, the cells were incubated with V450-conjugated anti-CD11c mAb (BD Biosciences, San Diego, CA, USA), PE-cy7-conjugated anti-CD80 monoclonal antibody (mAb) (eBioscience, San Diego, CA, USA), PE (phycoerythrin)-cy7-conjugated anti-CD86 (eBioscience), and PE-conjugated anti-MHC class I and II mAbs (eBioscience) for 30 min. The fluorescence was measured using flow cytometry (FACSCanto, BD Biosciences), and the data were analyzed using Flowjo data analysis software (TreeStar, Inc., Ashland, OR, USA).

### 3.9. ELISA

We collected the cell culture supernatant and stored it at −80 °C until use. We determined the levels of TNF-α, IL-6, IL-12p70, and IL-10 by ELISA using a commercial reagent kit according to the manufacturer’s instructions.

## 4. Conclusions

We prepared SMNPs and IONPs and analyzed their physicochemical properties and subcellular localization by measuring the particle size and zeta potential using TEM. In addition, we compared the cytotoxicity of SMNPs and IONPs with that of iron citrate, which is the typical form of iron used in iron supplements. Surface modification prevented agglomeration of particles during the preparation of samples and during cell incubation. In addition, results of ICP-MS indicated that the SMNPs and IONPs may have undergone phagocytosis by the bone marrow cells during incubation. However, no interaction was observed between the bone marrow cells and iron citrate. The results of ICP-MS were similar to those of TEM, which showed that the bone marrow cells treated with IONPs had more iron particles. The effects of phagocytosis were stronger in IONPs than in SMNPs, and thus, SMNPs appeared to be more toxic than IONPs. We examined the cytotoxicity of nanoparticles using various cell lines. Our results showed that iron nanoparticles used at a concentration of 1% were not toxic to the bone marrow cells. In addition, they did not affect the expression of cell surface markers and LPS-induced secretion of cytokines by the BMDCs. Although many studies have been performed, further detailed studies are required to clarify the toxicity of various nanoparticles [14,15]. Our study provides basic information for understanding the interaction of nanoparticles with biological systems. Moreover, iron nanoparticles can be used safely in various nanomedicines such as nutrient supplements, contrast agents in magnetic resonance imaging, and drug delivery systems. Accurate evaluation of the toxicity of iron nanoparticles on the basis of their physicochemical properties can facilitate the application of nanoparticles in different new fields [23].

Determination of the toxicity of IONPs based on their physicochemical properties can enable the use of IONPs in various fields.
